# Botulinum toxin‐a treatment reduces human mechanical pain sensitivity and mechanotransduction

**DOI:** 10.1002/ana.24122

**Published:** 2014-03-13

**Authors:** Kathryn Paterson, Stéphane Lolignier, John N. Wood, Stephen B. McMahon, David L. H. Bennett

**Affiliations:** ^1^ Wolfson Centre for Age‐Related DiseaseKing's College LondonLondon; ^2^ Molecular Nociception GroupWolfson Institute for Biomedical ResearchUniversity College LondonLondon; ^3^ Nuffield Department of Clinical NeurosciencesUniversity of OxfordOxfordUnited Kingdom

## Abstract

The mechanisms underlying the analgesic effects of botulinum toxin serotype A (BoNT‐A) are not well understood. We have tested the hypothesis that BoNT‐A can block nociceptor transduction. Intradermal administration of BoNT‐A to healthy volunteers produced a marked and specific decrease in noxious mechanical pain sensitivity, whereas sensitivity to low‐threshold mechanical and thermal stimuli was unchanged. BoNT‐A did not affect cutaneous innervation. In cultured rodent primary sensory neurons, BoNT‐A decreased the proportion of neurons expressing slowly adapting mechanically gated currents linked to mechanical pain transduction. Inhibition of mechanotransduction provides a novel locus of action of BoNT‐A, further understanding of which may extend its use as an analgesic agent. Ann Neurol 2014;75:591–596

Botulinum toxin serotype A (BoNT‐A) is widely used in the treatment of conditions of muscle or glandular hyperactivity.^1^ Inhibition of vesicular release of acetylcholine is achieved by receptor‐mediated entry of BoNT‐A into presynaptic nerve terminals followed by cleavage of the 25kDa synaptosomal‐associated protein (SNAP‐25) required for presynaptic vesicular docking. BoNT‐A has recently been noted to have analgesic effects in animal models of inflammatory and neuropathic pain^2^ and clinically has efficacy in the treatment of headache^4^ and neuropathic pain.^5^

The mechanisms by which the analgesic effects of BoNT‐A are mediated are not fully understood. One mechanism is the inhibition of neurogenic inflammation by blocking neurotransmitter release from sensitized nociceptors, reducing peripheral sensitization.^6^ In vitro, BoNT‐A has been found to inhibit neuropeptide release,^7^ and BoNT‐A reduces the vascular response to algogens such as capsaicin applied to human skin.^8^ Other potential analgesic mechanisms include retrograde transport of BoNT‐A by sensory neurons and inhibition of neurotransmitter release by their central terminals.^9^ We have tested the hypothesis that BoNT‐A can have modality‐specific effects on sensory function through alterations in nociceptor transduction.

Our ability to perceive specific sensory modalities is critically dependent on the detection of the sensory stimuli by specialized populations of sensory neurons. The past 2 decades have led to a much clearer understanding of the molecular and biophysical processes underlying sensory transduction.^10^ This is mediated by ion channels and G‐protein–coupled receptors expressed at neuron terminals and activated by specific stimuli. The pattern in which these molecules are expressed determines the response of sensory neurons to particular thermal, mechanical, and chemical stimuli. The neural substrates underling different sensory modalities are distinct and as such can be manipulated independently both in disease states and by therapeutics.^11^ We first investigated whether treatment of humans with BoNT‐A resulted in altered perception of specific sensory modalities and found that intradermal administration of BoNT‐A to healthy volunteers selectively reduced mechanical pain sensibility.

The molecular identity of the ion channel that responds to noxious mechanical stimuli is not yet known; however, it can be studied electrophysiologically by measuring mechanically gated currents in rodent sensory neurons in culture. Such currents have rapidly adapting (RA) or slowly adapting (SA) kinetics underlying low‐threshold responses and mechanical nociception, respectively.^12^ In the second part of this study, we examine the effects of BoNT‐A on this transduction machinery to investigate the mechanism underlying the reduction in mechanical pain perception.

## Subjects and Methods

Healthy volunteers were recruited to take part in this study (12 male, 12 female; median age = 26 years, range = 21‐33 years). Each participant provided written informed consent prior to commencing each experiment in compliance with the declaration of Helsinki, and the protocol was conducted in accordance with the King's College London (BDM 10/11‐84) and National Health Service research ethical committees (11‐LO‐0159). Subjects underwent baseline quantitative sensory testing (QST) to the lateral lower leg 9cm above the lateral malleolus. Intradermal injections were given at 8 and 10cm above the lateral malleolus. One leg received two 200μl injections of 10U BoNT‐A (Xeomin) in 0.9% saline, and the other was injected with saline alone (the side that received BoNT‐A was randomized). Subsequent QST was performed at weekly intervals for 4 weeks in a double‐blinded fashion.

## QST

QST followed protocols of the German Research Network on Neuropathic Pain,^13^ including measurement of warm and cooling detection thresholds, cold and heat pain thresholds, mechanical detection threshold, mechanical pain threshold, and windup ratio (WUR; a measure of central facilitation of sensory processing). Following BoNT‐A injection, 4 weekly QST sessions were performed. Histamine iontophoresis (1% aqueous solution delivered using a 10‐second 1mA current) and topical 50% allyl isothiocyanate (AITC) were given as chemical challenges in the final week.^14^ Tracing onto acetate 10 minutes after histamine or AITC was used to calculate flare area.

**Figure 1:**
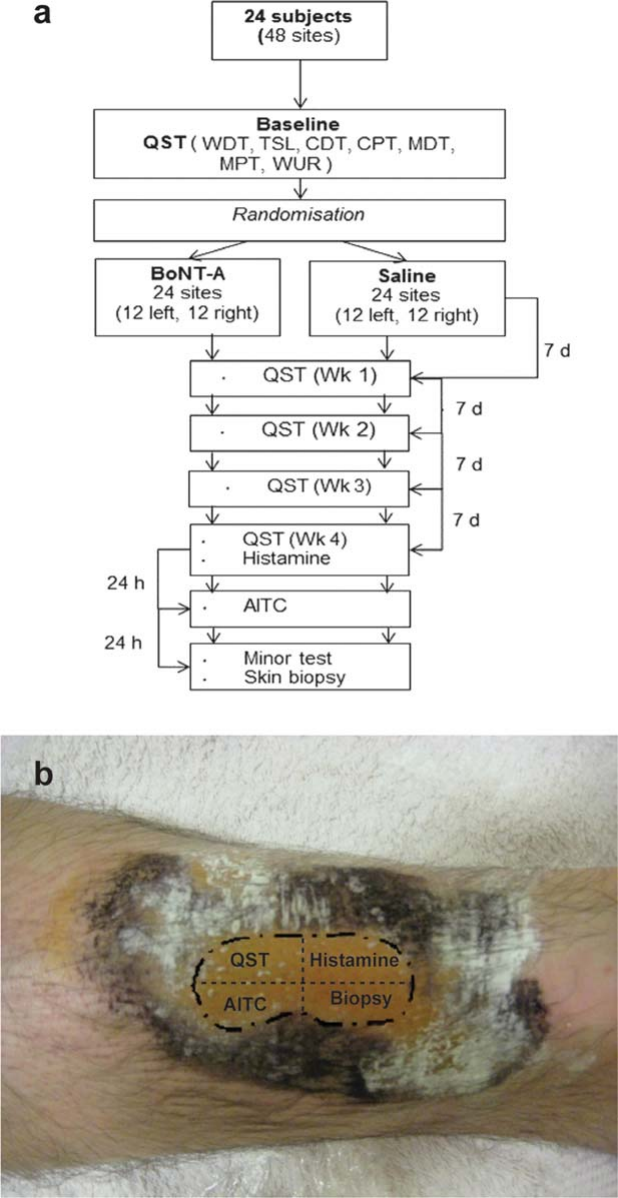
Psychophysics methodology. (A) Study design and sequence of psychophysical testing. AITC = allyl isothiocyanate; BoNT‐A = botulinum toxin serotype A; CDT = cold detection threshold; CPT = cold pain threshold; MDT = mechanical detection threshold; MPT = mechanical pain threshold; QST = quantitative sensory testing; TSL = thermal sensory limen; WDT = warm detection threshold; WUR = windup ratio. (B) Distal leg sites were tested bilaterally. Dotted area represents area of BoNT‐A treatment as revealed by the Minor sudomotor function test. This area and the corresponding contralateral area were divided into 4 sites as shown.

As a positive control, sudomotor function was tested using Minor's starch–iodine test^15^ to define the anhydrous area. Finally, 3mm skin punch biopsies were taken from vehicle and BoNT‐A–treated sites (Fig 1A). Skin was processed as previously described to determine intraepidermal nerve fiber density (IENFD).^16^

## Sensory Neuronal Cultures and Electrophysiological Recordings

Dissociated cultures of thoracic and lumbar dorsal root ganglions from 6‐ to 10‐week‐old C57Bl6/J mice and assessment of mechanically gated currents were performed as previously described.^12^ Ten units per milliliter BoNT‐A or the equivalent volume of saline was added to each dish immediately after culture, and cells were used for patch clamp studies 48 ± 4 hours after BoNT‐A treatment. Small dorsal root ganglion neurons (<30pF) were selected for electrophysiological recordings. KCl‐containing pipette solution was used for all recordings. Mechanical stimulation of cell bodies was achieved using a heat‐polished glass pipette controlled by a piezoelectric crystal drive (Burleigh LSS‐3000; Thorlabs, Newton, NJ). Two hundred fifty–millisecond mechanical stimulation steps were applied every 10 seconds in 1μm increments. Cells were considered as nonresponding when no current >10pA was observed in response to a 12μm stimulus. Responding cells were classified according to the adaptation kinetics of their currents (see Fig 3B).

**Figure 2:**
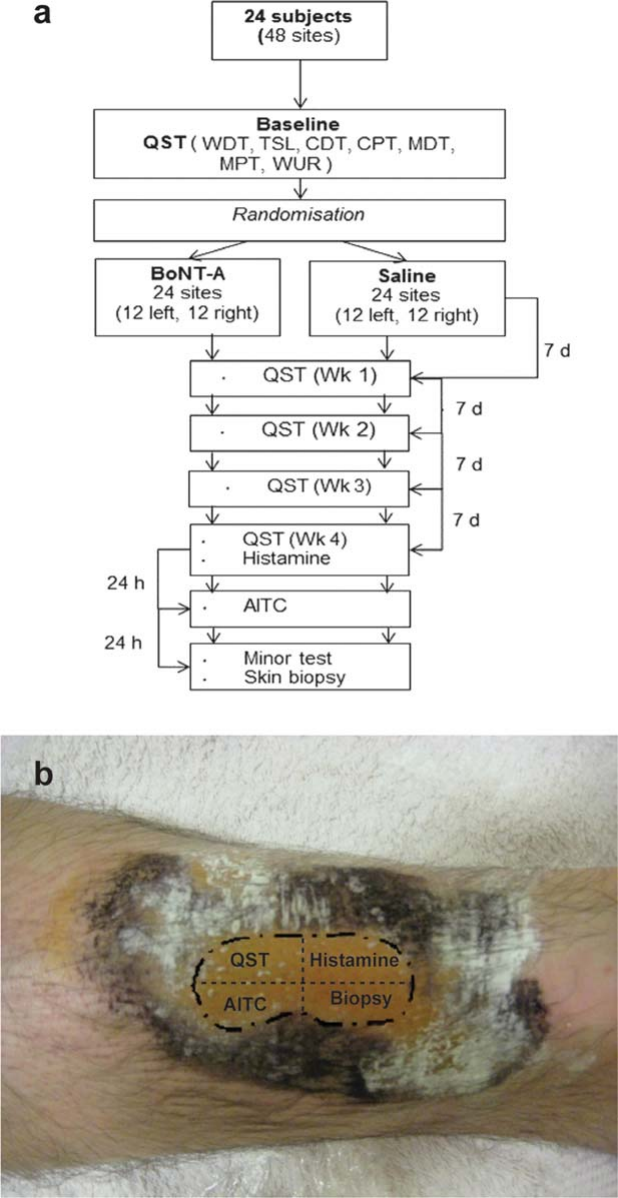
Mechanical quantitative sensory testing. Bilateral mechanical quantitative sensory testing was performed weekly. (A) No change was observed for mechanical detection threshold (MDT). (B) A significant increase in mechanical pain threshold (MPT) from week 2 was demonstrated in botulinum toxin serotype A (BoNT‐A)‐treated skin compared to saline control (week 2, *p* = 0.0361; week 3, *p* = 0.0065; week 4, *p* = 0.0261; paired *t* test). (C) Windup ratios remained unchanged. All data are represented as mean ± standard error of the mean, n = 24. **p < 0.05, ***p < 0.01.

## Data Analysis

All QST data were analyzed for each subject as a proportion of their baseline. Weekly QST data were analyzed using 2‐way repeated measures analysis of variance (ANOVA) with Bonferroni post hoc analysis. Current clamp data (firing threshold and resting potential) were analyzed using unpaired *t* test. Voltage clamp data were analyzed using the chi‐square test on raw cell numbers.

## Results

A schematic illustration of the study design is shown in Figure 1A. The starch–iodine test (Minor test) for sudomotor function confirmed adequate treatment with BoNT‐A in all cases (mean anhydrous area was 17.9 ± 2.4cm^2^; see Fig 1B). No subjects reported any side effects from the treatment of either leg. BoNT‐A treatment reduced histamine‐evoked itch and AITC‐evoked pain, and these changes were associated in both cases with reduced flare area (in both cases *p* < 0.05 BoNT‐A–treated skin compared to saline). Sensory thresholds were determined at baseline and for 4 consecutive weeks following injection of BoNT‐A or saline. There was no change in the ability of subjects to detect innocuous tactile stimuli (mechanical detection threshold) over the study period (Fig 2). Mechanical pain thresholds, however, were significantly increased in the BoNT‐A–treated region compared to the vehicle control area (*p* < 0.001, BoNT‐A vs saline, 2‐way repeated measures ANOVA). The WURs were unchanged between treatment areas. This is a psychophysical measure of hypersensitivity due to temporal summation within the central nervous system, and although a lack of effect on WUR cannot completely exclude a central effect of BoNT‐A, it led us to hypothesize that BoNT‐A could directly impair the transduction of mechanical stimuli at the periphery. In contrast to mechanical pain, we found that thermal detection and pain thresholds were not significantly different when comparing BoNT‐A–treated and saline‐treated skin (data not shown). Topical treatment with certain agents such as capsaicin cause altered sensory thresholds due to local sensory axon degeneration.^17^ However, there was no difference in IENFD (*p* = 0.7470, paired *t* test, BoNT‐A vs saline) following BoNT‐A treatment, and morphologically the fibers appeared normal. This suggests that the reduction in mechanical pain sensitivity was due to a functional effect of BoNT‐A on mechanosensitive nociceptors. Because BoNT‐A selectively increased mechanical pain threshold but did not alter thermal detection or pain thresholds, we investigated the impact of BoNT‐A on mechanotransduction.

**Figure 3:**
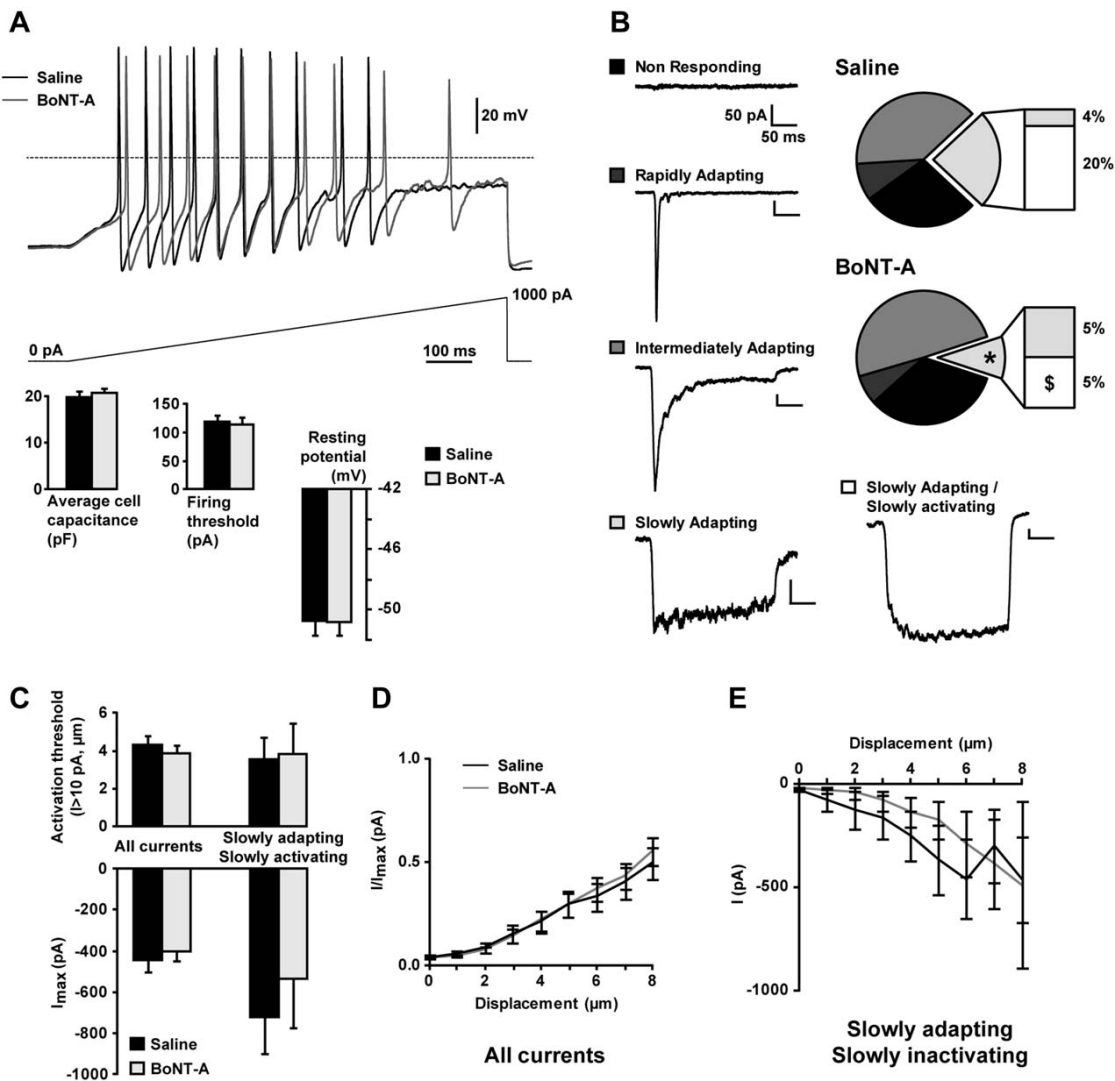
Excitability and mechanically activated currents in botulinum toxin serotype A (BoNT‐A)‐treated sensory neurons. (A) Excitability of small sensory neurons (<30pF) cultured for 48 hours in the presence of 10U/ml BoNT‐A or saline was assessed by current clamp recordings. Representative firing recorded in neurons in response to the injection of a ramp current of 1nA over 1 second is shown. Bar graphs showing average capacitance, firing thresholds, and resting potentials are presented as mean ± standard error of the mean. No significant differences were found (unpaired *t* test, n = 46 saline, n = 55 BoNT‐A). (B) Mechanically gated currents were subsequently recorded in the same neurons in voltage clamp mode and distinguished by their adaptation kinetics. A significant reduction in the proportion of neurons showing a slowly adapting current was observed in the BoNT‐A–treated group (*p = 0.0410, chi‐square test). This decrease specifically affects a subpopulation of cells showing a slowly adapting/slowly activating current ($*p* = 0.0145 vs *p* = 0.3992 for slowly adapting/fast activating currents, chi‐square test). Representative traces of mechanically gated current types are shown as a legend. (C) Activation threshold and maximum intensity of mechanically activated currents in sensory neurons, taken together or considering the slowly adapting currents only. The activation threshold is given by the first stimulation intensity producing a current >10pA. No significant differences were found (unpaired *t* test, n = 46–55 for all currents, n = 11–6 for slowly adapting currents). (D, E) Current–displacement curves recorded from all neurons (D) or neurons showing a slowly adapting/slowly activating current only (E). As currents of different natures are pooled together in D, values were normalized by the maximum current intensity for each cell.

The effect of BoNT‐A on neuronal excitability and mechanotransduction was assessed at the cellular level in cultured mouse dorsal root ganglion neurons exposed to 10U/ml BoTN‐A for 2 days. BoNT‐A treatment had no effect on the overall neuronal excitability, characterized by the firing threshold in response to current injection, or on the membrane resting potential, as shown in Figure 3. RA and intermediately adapting mechanically activated inward currents were unchanged. However, there was a significant decrease in the proportion of cells expressing an SA mechanically gated current (11 of 46 saline, 6 of 55 BoNT‐A, *p* = 0.0410, chi‐square test) in response to local pressure applied to the cell soma. Further analysis showed that BoNT‐A treatment reduced by 3.5 fold the proportion of neurons expressing an SA current with slow activation kinetics (specifically, 9 of 46 saline, 3 of 55 BoNT‐A, *p* = 0.0145, chi‐square test). BoNT‐A had no effect on the activation threshold of mechanically gated currents, and only a slight decrease in the peak current intensity could be seen within the population of neurons showing slowly adapting currents. These results are consistent with our psychophysical observations, as the SA mechanically gated current found in dorsal root ganglion neurons has been linked to noxious mechanosensation.^12^

## Discussion

This study was designed to investigate the modality‐specific effects of intradermal injection of BoNT‐A on human sensory function. The BoNT‐A treatment regime reduced itch and pain sensibility in response to challenge with histamine and AITC, respectively. In both cases neurogenic flare was reduced, consistently with the hypothesis that BoNT‐A reduces neurogenic inflammation and in agreement with previous studies.^6^ We also found that BoNT‐A selectively reduced the subject's sensitivity to noxious mechanical stimuli, but there was no change in response to low‐threshold mechanical stimuli or to thermal stimuli. The lack of effect of BoNT‐A on baseline thermal sensitivity is in agreement with other studies.^18^ The effects of BoNT‐A on mechanosensation in naive human skin over this long time course has not previously been determined. One study found no effect of intracutaneous BoNT‐A on mechanical pain threshold; however, the longest time point at which this was assessed was at 48 hours postinjection. Our data would suggest that this is not an optimum time window to detect change, given that the maximal effect we observed was at week 3 postinjection.^19^ The increased mechanical pain threshold is likely to represent changes in cutaneous nociceptive afferents; we were careful to inject the BoNT‐A into the dermis, and the mechanical probes used to assess mechanical pain thresholds selectively activate these cutaneous afferents.^20^ BoNT‐A injected into muscle (for instance in the treatment of disorders of motor hyperactivity) may exert its effects on muscle afferents by distinct mechanisms. BoNT‐A has been reported to impair neurite outgrowth in sensory neurons,^21^ but we did not observe any change in innervation density in BoNT‐A–treated skin. The WUR, which provides a psychophysical correlate of temporal summation leading to central sensitization, did not change following BoNT‐A treatment, and so we investigated whether BoNT‐A could alter peripheral mechanotransduction.

BoNT‐A had no effect on sensory neuronal excitability or on the proportion of neurons expressing transient mechanically gated currents, but significantly decreased the proportion of neurons expressing an SA mechanically gated current. Neurons expressing this SA mechanically gated current are believed to be involved in mechanonociception, as blockade of the channel responsible for this current by the conotoxin NMB‐1 increases mechanical pain thresholds, without affecting light touch or heat sensitivity.^12^ The channel(s) responsible for this current have yet to be identified. The TRPA1 channel is required for the generation of an SA current in a subset of peptidergic dorsal root ganglion neurons,^22^ and this correlates with functional changes in sensory terminals that demonstrate reduced action potential firing in response to noxious peripheral mechanical stimulation following TRPA1 blockade.^23^ However, TRPA1 is not sufficient for mechanotransduction, as it is unable to generate mechanically gated currents when expressed alone in a heterologous system.^22^

Owing to its delayed and chronic effects in humans, and the finding that BoNT‐A in vitro decreased the proportion of mechanosensitive cells showing slow currents, a direct pharmacological action on mechanosensitive channels of the toxin is unlikely. A more plausible possibility is an effect of BoNT‐A on the trafficking of mechanosensitive channels. In sensory neuron cultures, it has been shown that the upregulation of mechanically gated currents by inflammatory mediators acting through protein kinase C activation is suppressed by blocking vesicle trafficking with tetanus toxin.^25^ As more advanced preparations of BoNT‐A are produced, such as chimeric molecules that bind to nociceptors more effectively,^26^ and methods for transdermal delivery are improved,^27^ the applications of BoNT‐A as an analgesic may be extended. One potential implication of our findings is that patients with mechanical hypersensitivity (for instance following traumatic nerve injury or stump pain following limb amputation) may be more likely to respond to BoNT‐A. Insight into the effects of BoNT‐A on mechanotransduction, coupled with the molecular identification of the trafficked mechanotransducing apparatus, should aid in the optimization and assessment of the analgesic actions of BoNT‐A.

## Authorship

K.P. and S.L. are joint first authors.

## Potential Conflicts of Interest

D.L.H.B.: grants, Pfizer; member, Innovative Medicines Intitative Europain (public–private partnership between EU and EFPIA; industry members are Astellas, AstraZeneca, Pfizer, Esteve, UCB, Sanofi‐Aventis, Grünenthal, Eli Lilly, Neuroscience Technologies, and Boehringer Ingelheim).
